# Large-Scale Web Scraping for Problem Gambling Research: A Case Study of COVID-19 Lockdown Effects in Germany

**DOI:** 10.1007/s10899-023-10187-1

**Published:** 2023-01-27

**Authors:** Elke Smith, Simon Michalski, Kilian H. K. Knauth, Kai Kaspar, Nils Reiter, Jan Peters

**Affiliations:** 1 Department of Psychology, Biological Psychology, University of Cologne, Cologne, Germany; 2 Department of Psychology, Social and Media Psychology, University of Cologne, Cologne, Germany; 3 Department of Digital Humanities, University of Cologne, Cologne, Germany

**Keywords:** Problem gambling, Online gambling, COVID-19, Lockdown, Gambling forum

## Abstract

The COVID-19 pandemic and the measures to prevent its spread have had a negative impact on substance use behaviour. It is likely that social distancing and lockdown measures have also altered gambling behaviour, for instance shifting from land-based to online gambling. We used large-scale web scraping to analyse posting behaviour on a major German online gambling forum, gathering a database of more than 200k posts. We examined the usage of different subforums, i.e. terrestrial, online gambling and problem gambling sections, and changes in posting behaviour related to the casino closures that were part of the nationwide restrictions in Germany in 2020. There was a marked increase in newly registered users during the first lockdown compared to the preceding weeks, an increase in the number of posts in the online gambling subforum and concurrent decrease in the terrestrial gambling subforum. Further, the number of short-latency replies was higher during the first lockdown compared to the preceding weeks. Many users who posted in both the online and terrestrial forum contributed at least once to the problem gambling subforum, implying that the topic of problem gambling is widely discussed. Our findings may indicate a shift from terrestrial to online gambling during lockdown, and mirror the general increase in screen time and usage of online platforms after the onset of the COVID-19 pandemic. The analyses help to identify lockdown-related effects on gambling behaviour. These potentially detrimental effects pose a special threat for individuals at risk and may require monitoring and special public health measures.

## Introduction

### The COVID-19 Pandemic and Gambling Behaviour

The worldwide spread of the coronavirus SARS-CoV-2 starting in December 2019 and the resulting COVID-19 pandemic constitutes a substantial psychological stressor: due to repeated lockdowns, social interactions and everyday behaviours are affected and regulated in unprecedented ways (Wu et al., 2022). This is paired with high uncertainty regarding how the pandemic will evolve and how it will affect individuals. School and day care closures and job insecurities put additional strain on families (Martinsone & Tzivian, 2021). Accordingly, early meta-analyses suggest high levels of COVID-19-related psychological stress (Cooke et al., 2020). The COVID-19 pandemic and the associated containment measures put in place have had a negative impact on substance use behaviour (Boschuetz et al., 2020; Killgore et al., 2021; Vanderbruggen et al., 2020), very likely a consequence of heightened stress, social isolation and feelings of loneliness. It is reasonable to assume that this might also apply to gambling behaviour. In substance use disorders, stress is known to trigger craving and relapse (Hellberg et al., 2019; Mantsch et al., 2016; Sinha et al., 2007). Along similar lines, the severity of problem gambling is associated with stress (Elman et al., 2010; Loo et al., 2011).

The public on Twitter views the COVID-19 pandemic as a special threat to certain individuals, being more exposed to online gambling content (Fino et al., 2021). In the context of leisure activities, there was a marked change in the use of communication and social media during the lockdown periods (Meier et al., 2021). COVID-19 and the lockdown and social distancing measures put in place may therefore also alter gambling behaviours (e.g., behaviour might shift from terrestrial to online gambling). A study from a Canadian sample found that some individuals switched from terrestrial to online gambling during the COVID-19 pandemic and that especially high-risk gamblers exhibited an increased likelihood of online gambling behaviour (Price, 2020). An increase in online gambling during the COVID-19 quarantine period was reported in a dataset from an online study with mainly UK and US participants (Sallie et al., 2021). In contrast, reduced online gambling behaviour (online sports betting) was reported after COVID-19 measures were put in place for a dataset from Sweden, Germany, Finland, and Norway (Auer et al., 2020). A study from an American sample observed reduced online gambling, but increased substance use (Xuereb et al., 2021). In that study, only a minority of individuals who gambled substituted terrestrial with online gambling. However, these appeared to be vulnerable individuals with symptoms of problematic gambling behaviour. In a sample of Swiss terrestrial gamblers, no changes in online gambling participation during lockdown in Switzerland were found, but those who gambled online during lockdown played more often and longer than before (Lischer et al., 2021).

Changes in gambling behaviour caused by COVID-19 and the measures put in place have not been extensively studied to date and appear to heavily depend on the population or type of gambling activity studied. The effects of the COVID-19 pandemic on gambling behaviour likely depend on complex local and individual factors, including shutdowns of gambling venues and limited availability of sports betting due to cancellation of events (Auer et al., 2020; Gainsbury et al., 2021). In Germany, terrestrial gambling facilities have remained closed over longer periods of time due to lockdowns, which may have led to a shift from terrestrial to online gambling in certain individuals (see Appendix, Table 3). Importantly, the vast majority of the studies cited above rely on self-reports to quantify changes in gambling behaviour associated with COVID-19 measures. However, self-reports are subject to various biases, including social desirability (Krumpal, 2013) and recall bias (Bradburn et al., 1987), whereby computer-based surveys appear to yield significantly more reporting of socially undesirable behaviours compared to equivalent paper-based surveys (Gnambs & Kaspar, 2015). Therefore, the field might benefit from more objective measures of changes in gambling behaviour associated with different societal stressors or policy changes.

### Gambling-Specific Online Communities

The rise of social media use in conjunction with advances in machine learning, text mining and modelling have created novel opportunities for the use of social media “big data” to address research questions in psychology (Conway & O’Connor, 2016; Landers et al., 2016). One area that has attracted particular attention concerns the association between mental health problems and social media behaviour, e.g., with respect to the type of content posted on social media sites (Chancellor & De Choudhury, 2020; Merchant et al., 2019). In this line of work, for example, substance use was linked to posting behaviour on Twitter©(Cavazos-Rehg et al., 2015; Glowacki et al., 2021; Lamy et al., 2016). Also, some studies analysed gambling-related content on Twitter© created by online gambling operators (Killick & Griffiths, 2020) and users (Fino et al., 2021). While general social media sites such as Twitter© continue to be widely used, more extensive discussions of specific and potentially problematic activities (such as gambling) often occur in focused online communities. In the context of the COVID-19 pandemic, social media data have been leveraged to examine pandemic-related mental health problems and challenges, for instance with respect to addiction (Glowacki et al., 2021) and gambling behaviour more specifically (Fino et al., 2021).

Online gambling communities typically constitute discussion boards or internet forums where users share or discuss gambling experiences, strategies or gambling problems (Griffiths, 2010; Sirola et al., 2021). Survey studies revealed that high levels of engagement in online gambling communities occur more frequently in players suffering from higher levels of problematic gambling behaviour (Sirola et al., 2018, 2019). For example, amongst participants in a survey amongst Finnish gamblers (Sirola et al., 2018), 54.33% of online gambling forum users reported South Oaks Gambling Screen (SOGS) (Lesieur & Blume, 1987) scores> 2 (“some problems with gambling”), whereas this was the case for only 15.58% of respondents who reported to have never visited such a community site (Sirola et al., 2018). Studying such online communities might therefore yield insights into problematic gambling behaviour (Griffiths, 2010).

Building upon initial findings on changes in gambling behaviour during lockdown phases following the onset of the COVID-19 pandemic (Price, 2020; Sallie et al., 2021), our aim was to capture such changes through changes in gambling-related online communication patterns. Changes in actual gambling behaviour during the lockdown were difficult to measure, since the closure of gambling venues meant that no systematic investigation could take place on site. A systematic and objective measurement of online gambling behaviour was also hardly possible, since there are countless gambling providers and platforms that individual users visit. For this reason, we used large-scale web scraping to collect large amounts of publicly accessible data from a major German gambling website and looked at changes in posting behaviour related to the COVID-19 lockdown measures in Germany in 2020 as an indicator of possible changes in actual gambling behaviour. While posting behaviour on a gambling website certainly represents an indirect measure of actual gambling behaviour, it may still be a more objective measure of behaviour compared to self-reports, which have known shortcomings such as the social-desirability bias (Krumpal, 2013) and recall bias (Bradburn et al., 1987).

Since we retrieved a large data base of> 200k posts, a manual analysis of the forum posts was clearly not feasible. We therefore analysed engagement in the online community based on the relative use of different subforums, overall posting frequency, and potentially event-related changes in posting behaviour. We first describe the data set in detail. In a second step, we report analyses of posting behaviour related to the casino closures that were part of the lockdown measures in Germany. During the first lockdown period in Germany starting in March 2020, all “non-essential” facilities and businesses were closed, including schools and universities, restaurants, bars, clubs, and casinos. Citizens were asked to reduce social contacts to an absolute minimum and spending time in public was only permitted for small groups. A period with reduced incidence rates and re-openings in summer 2020 was followed by rising incidence rates of COVID-19 in fall 2020, and a second lockdown was instated in November, during which casinos and other “non-essential” facilities were closed again. We hypothesised that the nationwide restrictions in Germany following the COVID-19 outbreak, which included venue closures, would be reflected in the form of changes in the statistical properties of the posts, such as frequency and distribution across the subforums, compared to a preceding reference period.

## Methods

### Description of the Discussion Board

The discussion board is part of a German-speaking online casino and gambling website. We use the following terms to describe the discussion board: A *post* is a single text from an individual user. A *user* is a person with an account (profile page) who may write posts. An *initial post* is a post that is not a response to another post. A *reply* is a post that is created in response to another post. A *thread* constitutes an initial post plus all of its replies. Threads are grouped in eleven superordinate board topics, which centre around online casinos, slot machines, games such as roulette, blackjack and poker, gambling and gambling addiction. In order to compare posts related to online and terrestrial gambling, only subforums and, in some cases, individual subcategories of subforums that clearly state in their description that only online or terrestrial gambling topics may be posted there, are considered for the analyses. The *online subforum* therefore contains exclusively online gambling sections and the *terrestrial subforum* exclusively terrestrial gambling sections. Sections of the forum for which it is unclear whether the content is associated with online or terrestrial gambling are therefore not included in the online and terrestrial subforum, respectively. The *problem gambling subforum* refers to the problem gambling board topic and does not overlap with the online or terrestrial subforums. This subforum was included in the analysis due to the potential clinical relevance of its use.

A single post consists of a headline and content. Each post has a publication date, an author (user name), a designated position in the thread, a count of likes, and an indication whether the post constitutes a reply to another post. Posts may also be empty (post deleted if the content contained insults or topic closed by the board administrators).

A *regular* user has a unique profile page. A user may also be flagged as *blocked*, *anonymous* or *deleted*, in which case no unique user name or profile page is displayed or accessible. A single user has a unique author name, a status (e.g., *starter* and *verified* user), a count of posts, and a date of joining the discussion board. Newly registered users have the status *starter* for a certain period of time, depending on their activity. To become a verified user, a photo of the face and a sheet of paper with the user name and date must be uploaded.

We classified the users as belonging to one of the following user types: *Online only* (at least one post in the online gambling subforum and zero posts in other sections of the forum), *terrestrial only* (at least one post in the terrestrial gambling subforum and zero posts in other sections of the forum), *mixed* (at least one post in the online gambling subforum, at least one post in the terrestrial gambling subforum and zero posts in the problem gambling subforum), *online only + PG* (same as online only, but additionally at least one post in the problem gambling subforum), *terrestrial only + PG* (same as terrestrial only but additionally at least one post in the problem gambling subforum), *mixed + PG* (same as mixed but additionally at least one post in the problem gambling subforum) and *PG only* (at least one post in the problem gambling subforum and zero posts in other sections of the forum). Users that do not fit in any of the above categories are classified as *other*.

### Data Collection

The website was scraped in April 2021 using *Python* (version 3.6.9) and the *Requests* (version 2.18.4) and *Beautiful Soup* (version 4.6.0) libraries for *Python*. The obtained XML data were parsed into a relational *SQLite* database using *Python* and the *sqlite3* module for *Python*.

### Legal Aspects

Web scraping for scientific purposes is generally legal, as long as no access restrictions are circumvented (Klawonn, 2019). While users hold copyright to their posts, the German Copyright Act *UrhG* allows text and data mining. The data scraped from the website were generally accessible and technical measures designed to prevent web scraping were not disregarded. The scraped public user profiles do not contain personal information that would allow an individual person to be identified. The research conducted on the basis of the data serves non-commercial purposes only and the published results do not allow identification of natural persons.

### Definitions of the Lockdown Periods

**Fig. 1:**
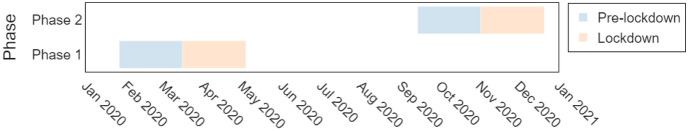
COVID-19 lockdown phases in Germany in 2020. The pre-lockdown periods are defined as the seven weeks before the respective lockdown phases

Closures of gambling halls in the federated states of Germany commenced between March 14 and 19 in 2020. Venues re-opened between May 4 and June 15th in 2020. The first lockdown was therefore defined as the period from Monday, March 16 to Sunday, May 3 of 2020, to enable an analysis of whole weeks. The first pre-lockdown period corresponds to the seven weeks before the first lockdown period. The second lockdown was then defined as the period from November 2 of 2020 to December 20 of 2020, to obtain a period of equal length as the first lockdown (the gambling halls were simultaneously closed down on November 2 of 2020 in all federated states of Germany and reopened in spring 2021). The second pre-lockdown period corresponds to the seven weeks before the second lockdown period. The lockdown phases in Germany in 2020 are depicted in Fig 1, for an overview on the casino closure periods in the federated states of Germany, see Appendix, Table 3.

### Modelling of Reply Latencies

User engagement may not only be reflected in the overall number of posts and replies, but also in the timing of user behaviour. To address this issue, for each of the four pre-defined periods (pre-lockdown 1, lockdown 1, pre-lockdown 2, and lockdown 2), we modelled the temporal evolution of reply frequencies following an initial post. We only included replies within the first 7 days following the initial post, and only considered initial posts up to 7 days before the end of the respective period to ensure that all considered reply posts fall within the respective period. The reply latencies were binned into bins of 8 h (resulting in 21 bins in total, ranging from 0 to 167 h for a complete week). We modelled the reply latencies as exponential decay with

$$\begin{aligned} y(0) = y0 - a * e ^ {-k * x} + a \end{aligned}$$

were *y0* is the intercept, *a* the asymptote and *k* the decay rate. The parameter distributions were estimated via *Hamiltonian Monte Carlo* sampling (4 chains, 4000 samples, warmup = 2000, thinning = 2) using *Stan* (2.21.0), and the *Python* interface to *Stan* *PyStan* (2.19.1.1). Chain convergence was determined such that $\hat{R}$ $\le$ 1.01 (Gelman & Rubin, 1992). We report directional Bayes factors (*dBFs*) (Kass & Raftery, 1995; Marsman & Wagenmakers, 2017) to quantify the degree of evidence for reductions and increases, respectively, in parameter values for *y0*, *a* and *k* for the pre-lockdown and lockdown phases via kernel density estimation in Python. The *dBFs* are defined as the ratio of the integral of the posterior distribution from $- \infty$ to 0 versus the integral from 0 to $\infty$. For instance, a *dBF* of 10 indicates that a positive directional effect is ten times more likely than a negative directional effect.

## Results

We first describe the dataset, before focusing on the specific analyses of COVID-19 lockdown-related effects.

### Characteristics of the Discussion Board

The discussion board is divided into eleven superordinate board topics. In total, there were 205,385 single posts, pertaining to 7,902 threads and 4,428 registered user profiles (excluding anonymous and blocked users whose number cannot be determined). Anonymous and blocked users wrote 46,100 of the 205,385 posts.

#### Users

**Fig. 2:**
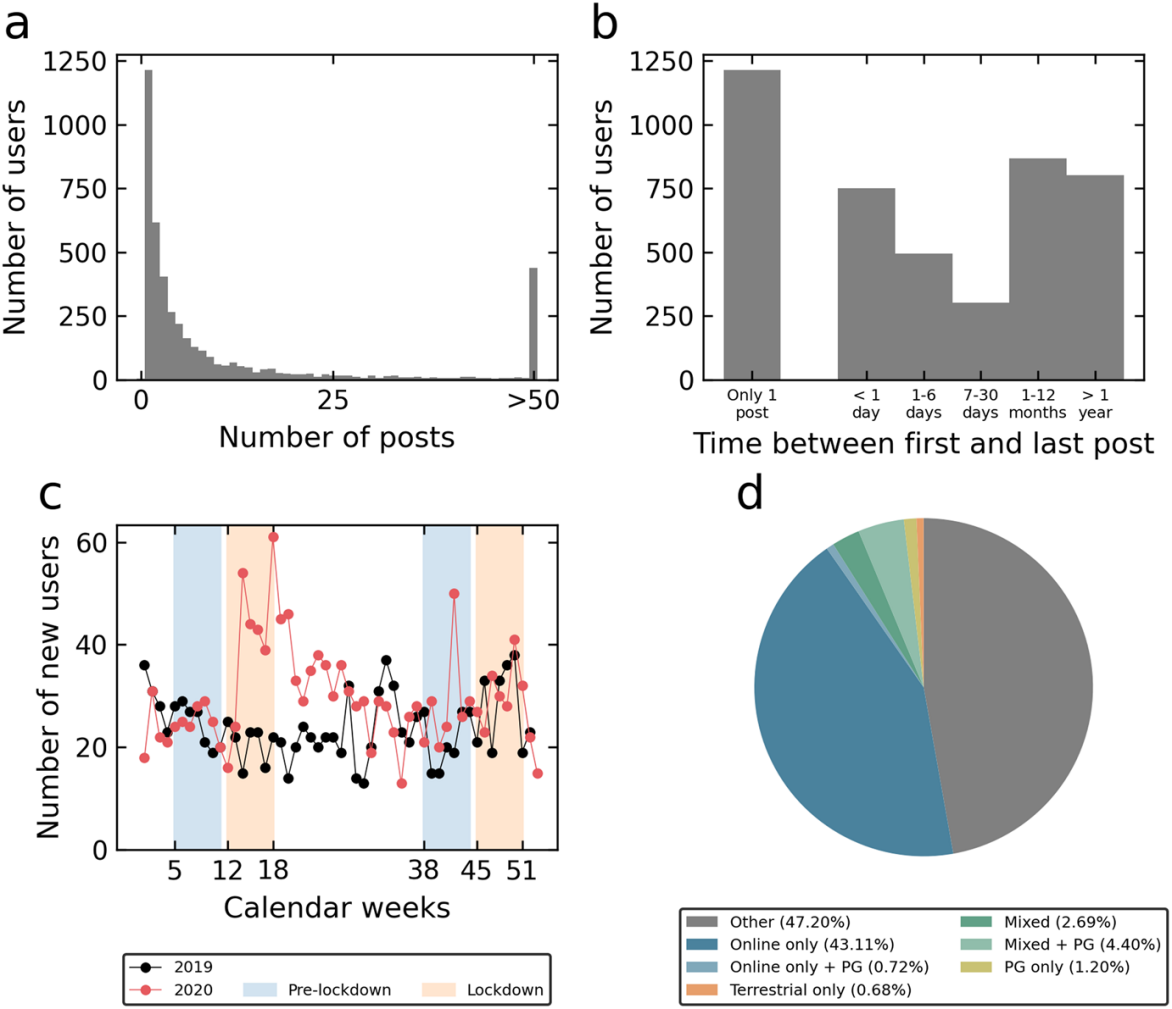
**a** Distribution of posts and users; **b** Time between first and last post; **c** Number of newly registered users per year, calendar week and phase (pre-lockdown and lockdown), **d** Proportion of user types. Zero users were classified as *terrestrial only* *+ PG*. *PG* problem gambling

On average, 36.00 days (*SD* = 125.24) passed between registration on the platform and the first post. 1552 users were flagged as *starter* (35.05% of all users), and 181 as *verified* users (4.09% of all users). There were on average 35.94 posts per user (*SD* = 168.81). Most users (3272 users or 74% of all users) published between 0 and 10 posts (see Fig. 2a). Many users posted only once (*N* = 1214; 27.42% of all users), or wrote several posts within a day (*N* = 751; 16.96% of all users), and then no more. However, there are also many users who are active in the forum over a longer period of time, for which the time between the first and the last post is 1 year (*N* = 867; 19.58% of all users) or more (*N* = 802; 18.11% of all users) (see Fig. 2b). The 100 most active users (2.26% of all users) contributed to 42.92% of the posts. There was a marked increase in newly registered users during the first lockdown phase in 2020 (see Fig. 2c). There were 175 new registrations during the first pre-lockdown phase in 2020, in comparison to 281 new registrations during the first lockdown phase, which represents an increase of 60.57%. During the second pre-lockdown period there were 199 new registrations, compared to 215 new users during the second lockdown period (increase of 8.04%). A large proportion of users were classified as *online only*, i.e. had at least posted once the online gambling subforum, but not in other subforums (see Fig. 2d). About half of the users did not fit into any of the predefined categories (classified as *other*, see section 2.1). Among the less frequent user types, the most frequent category was *mixed + PG*. Only very few users posted exclusively in the problem gambling subforum or terrestrial gambling subforum.

#### Posts

**Fig. 3:**
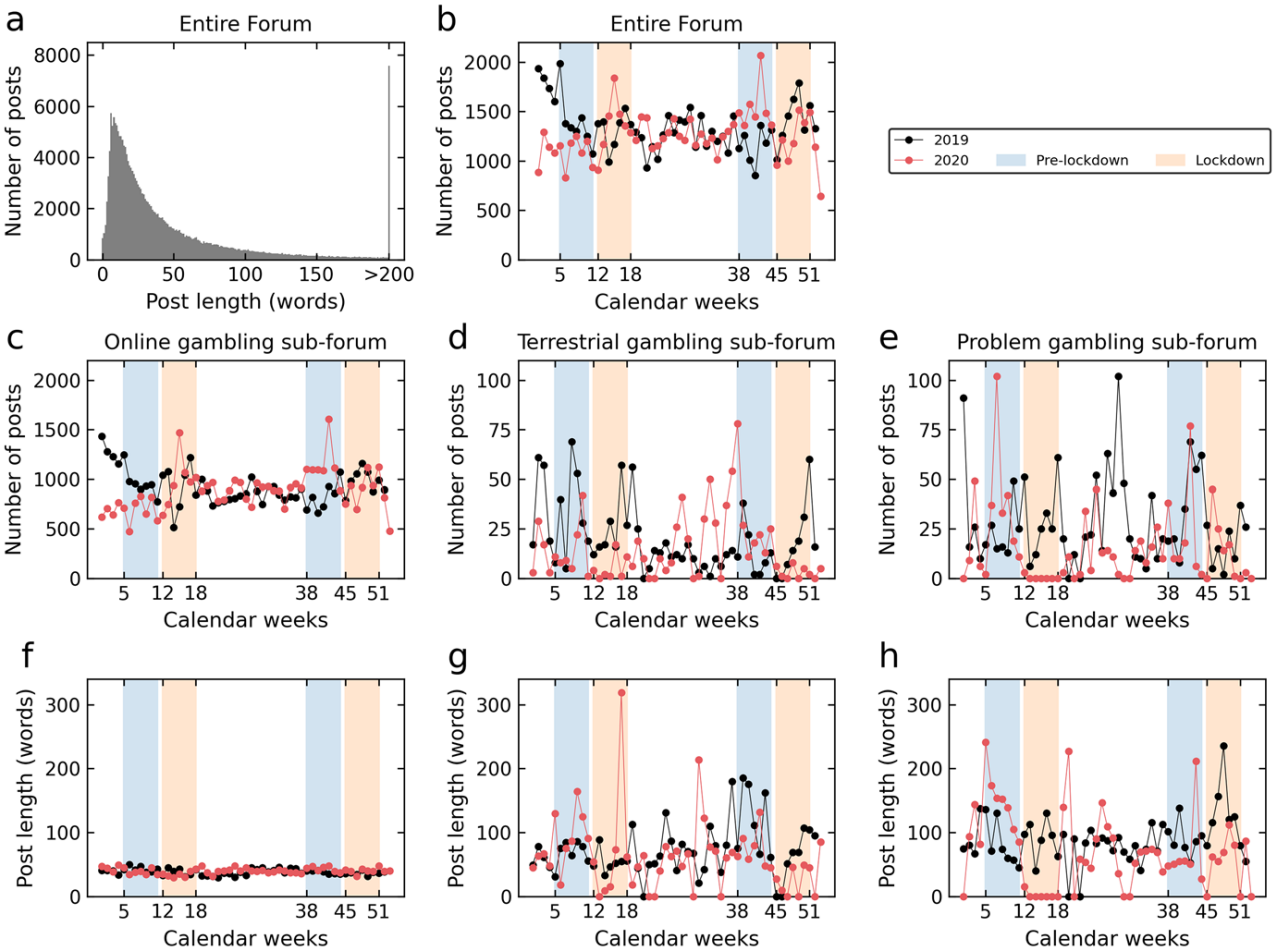
Distribution of posts for the entire forum and for the subforums. **a** Distribution of post length and number of posts; **b** Number of posts per year, calendar week and phase (pre-lockdown and lockdown) for the entire forum; **c**–**e** Number of posts per year, calendar week and phase (pre-lockdown and lockdown) for the online gambling, terrestrial gambling and problem gambling subforum, respectively; **f**–**h** Post length per year, calendar week and phase (pre-lockdown and lockdown) for the online gambling, terrestrial gambling and problem gambling subforum, respectively

The online gambling subforum contained the most posts (*N* = 135,785; 66.11% of all posts), followed by the problem gambling subforum (*N* = 4150; 2.02% of all posts), and finally the terrestrial gambling subforum (*N* = 3365; 1.64% of all posts). The percentage of initial compared to reply posts was similar for the subforums (42.78% to 57.22% for the entire forum, 45.03% to 54.97% for the online gambling subforum, 41.60% to 58.40% for the terrestrial gambling subforum, and 41.40% to 58.60% for the problem gambling subforum). During the first lockdown compared to pre-lockdown period, the number of posts increased by 41.48% in the online gambling subforum, while total posts decreased by 63.27% in the terrestrial gambling subforum, and by 98.78% in the problem gambling subforum. In contrast, the number of posts decreased during the second lockdown period in the online (by 18.81%), in the terrestrial (by 88.66%) and in the problem gambling subforum (by 36.65%) compared to the corresponding second pre-lockdown periods (see Table 1 and Fig. 3c–e).

With regard to post length, on average (across all subforums), a post contained 50.12 words (*SD* = 76.44). Most posts contained 6 words and half of the posts were at least 27 words long (see Fig. 3a). Posts in the terrestrial gambling subforum were longer (*M* = 81.89, *SD* = 112.93) than in the online gambling subforum (*M* = 43.75, *SD* = 65.28). The problem gambling subforum contained, on average, the longest posts among all superordinate board topics (*M* = 100.73, *SD* = 127.78). During both the first lockdown compared to pre-lockdown period, post length decreased in all three types of subforums (see Table 1). However, while the post length decreased only slightly in the online gambling subforum (reduction of 10.64%), the average post length dropped markedly in the terrestrial gambling subforum (by 41.43%) and problem gambling subforum (by 89.63%). For the second lockdown compared to pre-lockdown phase, average post length dropped by 6.77% in the online forum, and by 45.11% in the terrestrial forum, but increased by 22.92% in the problem gambling subforum (see Table 1 and Fig. 3f–h).

**Table 1:** Mean number of posts and mean post length for each phase, for the entire forum and for the online, terrestrial and problem gambling subforums, respectively

		Entire forum	OG subforum	TG subforum	PG subforum
*N* posts	Pre-LD1 1	7628	4836	98	246
	LD 1	9489	6842	36	3
	Pre-LD 2	10783	7981	194	161
	LD 2	8737	6480	22	102
*M* post length (words)	Pre-LD 1	49.91	39.27	118.55	147.78
	LD 1	40.25	35.09	69.44	15.33
	Pre-LD 2	46.96	43.71	72.38	56.58
	LD 2	43.76	40.75	39.73	69.55

*OG* online gambling, *TG* terrestrial gambling, *PG* problem gambling, *LD* lockdown

### Reply Latencies

**Fig. 4:**
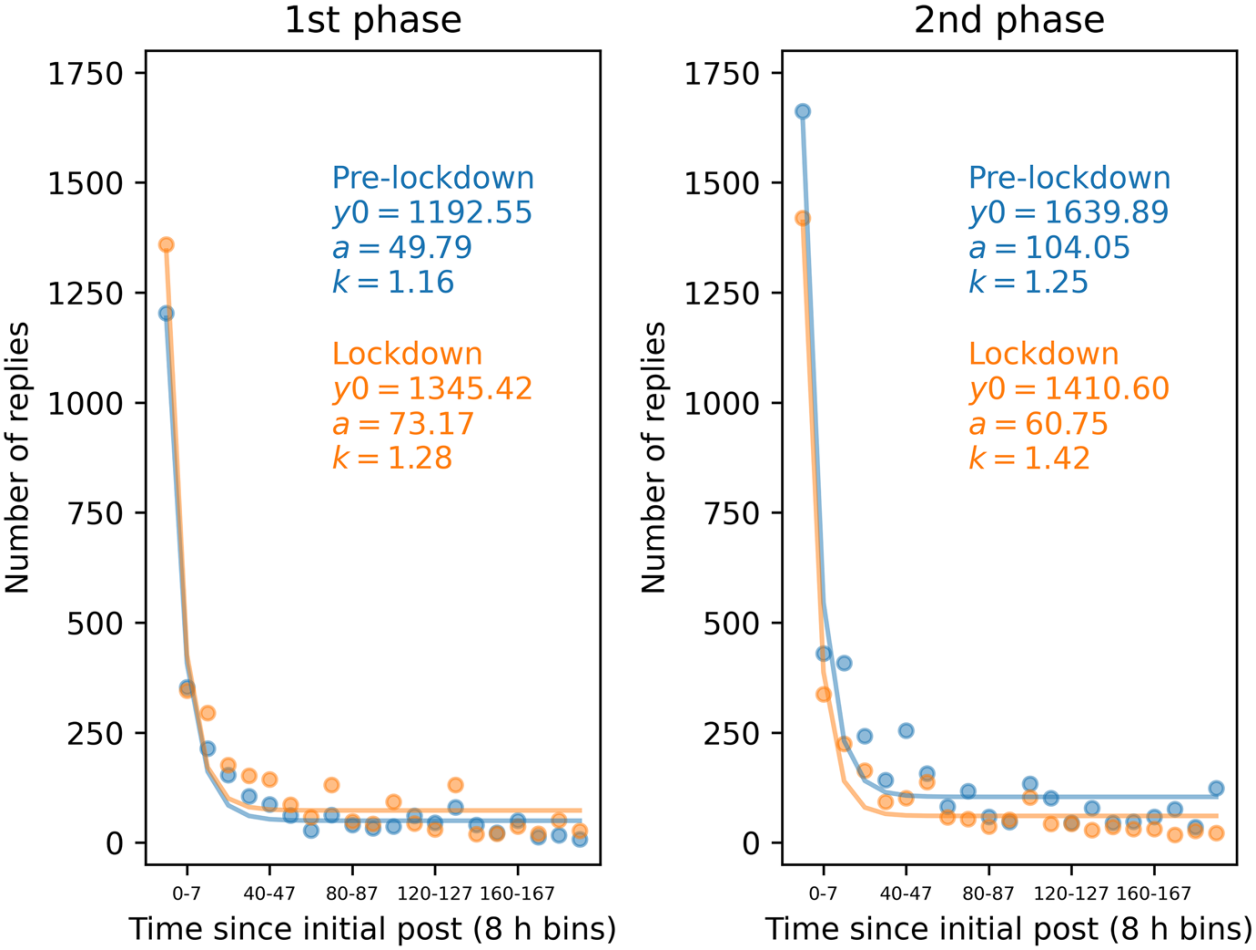
Modelled reply latency curves for the 4 phases overlayed onto the data. Reply latency (time since initial post) was binned into bins of 8 h. The latencies were modelled as exponential decay with *y0* intercept, *a* asymptote, and *k* decay rate. *Blue* pre-lockdown phases, *orange* lockdown phases (Color figure online)

**Fig. 5:**
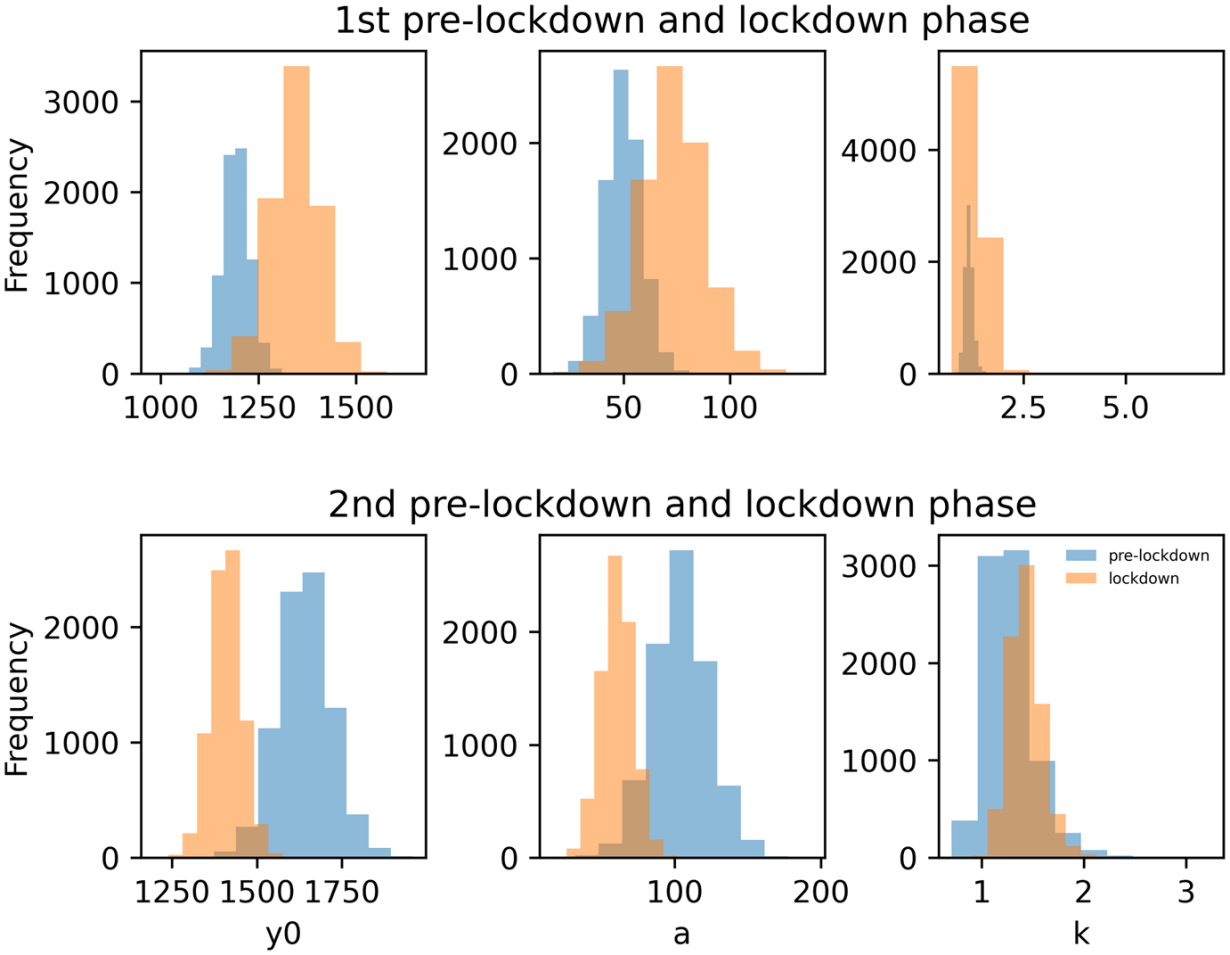
Posterior distributions of the latency model parameters for the pre-lockdown (blue) versus lockdown (orange) phases. The latencies were modelled as exponential decay with *y0*: intercept, *a*: asymptote, and *k*: decay rate (Color figure online)

For each of the phases, we modelled the relationship between reply frequency and latency, respectively, between initial posts and the replies that followed as an exponential decay function with offset *y0*, asymptote *a* and decay rate *k* (see Section 2.5). To this end, the reply latencies (elapsed time since initial post) were binned into bins of 8 h. The data and the modelled latency curves per phase (pre-lockdown and lockdown phase 1 in spring 2020, and pre-lockdown and lockdown phase 2 in winter 2020) are depicted in Fig. 4. The posterior distributions of the exponential decay model parameters are depicted in Fig. 5. The medians of the group-level posterior distributions were as follows: pre-lockdown 1: *y0* = 1192.55, *a* = 49.79, *k* = 1.16; lockdown 1: *y0* = 1345.42, *a* = 73.17, *k* = 1.28; pre-lockdown 2: *y0* = 1639.89, *a* = 104.05, *k* = 1.25; lockdown 2: *y0* = 1410.60, *a* = 60.75, *k* = 1.42. The medians and highest posterior density intervals of the difference distributions for the first and second phase for each of the three decay model parameters are listed in Table 2.

While the intercept *y0* and the asymptote *a* were higher during the 1st lockdown compared to the 1st pre-lockdown phase, the opposite was true for the 2nd phase. Here, *y0* and *a* were lower for the lockdown compared to pre-lockdown period. The decay rate *k* was higher for both lockdown compared to pre-lockdown phases. An increase during the first lockdown compared to pre-lockdown phase was 63 times more likely for the offset *y0* (*dBF* = 62.67), 10 times more likely for the asymptote *a* (*dBF* = 10.47), and 3 times more likely for the decay parameter *k* (*dBF* = 3.37). Comparing the second lockdown compared pre-lockdown phase, a decrease was 132 times more likely than an increase for the offset *y0* (*dBF* = 132.74), and 38 times more likely for the asymptote a (*dBF* = 38.91), while an increase was 3 times more likely than a decrease for the decay rate *k* (*dBF* = 3.23).

This indicates that the number of short-latency replies was higher for lockdown phase 1 compared to pre-lockdown phase 1, while the reverse was true for lockdown phase 2 compared to pre-lockdown phase 2. In the days following an initial post, the number of replies remained higher during lockdown phase 1 compared to pre-lockdown phase 1. Here, too, the reverse pattern emerged for lockdown phase 2 compared to pre-lockdown phase 2. The decline in the number of replies was similar for phases 1 and 2.

This indicates that the number of short-latency replies was higher for lockdown phase 1 compared to pre-lockdown phase 1, while the reverse was true for lockdown phase 2 compared to pre-lockdown phase 2. In the days following an initial post, the number of replies remained higher during lockdown phase 1 compared to pre-lockdown phase 1. Here, too, the reverse pattern emerged for lockdown phase 2 compared to pre-lockdown phase 2. The decline in the number of replies was similar for phases 1 and 2.

**Table 2:** Medians and 95% HDIs of the posterior difference distributions (lockdown minus pre-lockdown phase) for the parameters of the latency model

	Phase 1 (lockdown 1 - pre-lockdown 1)	Phase 2 (lockdown 2 - pre-lockdown 2)
*y0*	153.08 (17.89 to 298.24)	$-$230.86 ($-$408.76 to $-$44.28)
*a*	23.41 ($-$8.17 to 59.46)	$-$43.17 ($-$88.85 to $-$0.89)
*k*	0.12 ($-$0.30 to 0.61)	0.17 ($-$0.44 to 0.69)

*HDI* highest density interval, *y0* intercept, *a* asymptote, *k* decay rate

## Discussion

Using web scraping, we analysed changes in posting behaviour in a large German online gambling forum related to the casino closures in spring and winter of 2020 that were part of the nationwide restrictions in Germany to prevent the spread of COVID-19. We hypothesised that the restrictions, which included venue closures, would be reflected in the form of changes in the statistical properties of the posts, such as frequency and distribution across the subforums, compared to a preceding reference period.

### Description of the Discussion Board

Being part of an online casino and gambling website, the main topic of the discussion board is online gambling. The online gambling subforum makes up two-thirds of all forum posts, while the problem and terrestrial gambling subforum contain only a minority of all posts. However, the online subforum has the shortest posts, followed by the terrestrial gambling subforum. The problem gambling subforum contains the longest posts, which are on average twice as long as the average post. There is a substantial number of users who write a single post (27.42% of all users), or several posts within a day, but then do not continue posting (16.96% of all users). However, many users of the forum actively post over a longer period of time (> 35% of all users). The classification of user types shows that almost half of the users are active in the online gambling subforum only. Only a very small percentage of users posts exclusively in the problem gambling subforum or terrestrial gambling subforum. It is particularly striking that there was a marked increase in newly registered users during the first lockdown phase in 2020 compared to the weeks preceding the lockdown. Even if it is not possible to directly infer actual gambling behaviour from participation on online forums, however, this may well reflect a lockdown-induced shift from terrestrial to online gambling.

### Lockdown Effects

During the first lockdown phase in spring 2020, compared to the previous reference period, there was a substantial increase in newly registered users, which likely mirrors the general increase in social media and internet usage during lockdown reported in different samples from various countries (Gupta et al., 2021; Lemenager et al., 2021; Vall-Roqué et al., 2021). Further, there was an increase in the number of posts in the online gambling subforum with a concurrent decrease in the number of posts in the terrestrial gambling subforum. This shift from terrestrial to online likely reflects the effects of the casino closures that were part of the nationwide restrictions in Germany. Data from UK and Swiss samples show that decreases in terrestrial gambling coincided with proportionate increases in online gambling (Close et al., 2022; Emond et al., 2022; Lischer et al., 2021).

Modelling the relationship between reply frequency and latency between initial posts and the replies that followed for each of the phases allowed us to quantify the immediacy of user engagement with posts. This revealed that the number of short-latency replies (i.e. replies that were posted within 7 h after the initial post) was substantially higher during lockdown 1 compared to pre-lockdown 1. Furthermore, throughout the week following an initial post, the number of replies remained higher during lockdown 1 compared to pre-lockdown 1, as reflected in an elevated asymptote of the decay model. The increase in reply latencies during the first lockdown may reflect the general marked increase in screen time and/or usage of online platforms and media after the onset of the COVID-19 pandemic (Lemenager et al., 2021; Meier et al., 2021; Pandya & Lodha, 2021).

Interestingly, this pattern was reversed during the second lockdown. In contrast to the first lockdown, the number of posts in the online gambling subforum decreased during the second lockdown compared to the reference period. However, “baseline” numbers in the pre-lockdown phase were higher for the second compared to the first lockdown phase. Before the first lockdown in 2020, terrestrial gambling venues were likely visited as usual. However, the degree to which this was the case prior to the second lockdown period is unclear. While gambling venues were open during this time, social distancing measures were nonetheless in place, and players may have adapted their gambling behaviour due to the infection risk associated with terrestrial gambling. However, the number of posts in the terrestrial gambling subforum increased after the first lockdown and decreased again during the second pre-lockdown phase. Nonetheless, we cannot conclude from the data whether this actually reflects increased revisiting of gambling venues after the first lockdown. It would also be conceivable to have few visits being discussed very extensively in the forum. Also, the second lockdown had been announced for some time and was foreseeable, whereas the first lockdown was a condition never experienced before. Therefore, the second pre-lockdown phase is likely affected by a variety of additional factors which complicate a direct comparison of the first and second lockdown effects. The first pre-lockdown phase is likely a baseline that reflects “normal” gambling behaviour much more accurately than the second pre-lockdown phase.

Our analysis also examined posting behaviour in the problem gambling subforum, which might provide insights into potential lockdown-related effects on disordered gambling. It is particularly striking that the absolute number and length of posts in the problem gambling subforum dropped substantially during the first lockdown compared to the reference period. Perhaps problem gambling-related topics, such as relapse, recent problem gambling behaviour, tips on how to quit gambling as well as experiences with therapy, were less important for users to discuss about in the forum during the first lockdown. This change may be explained by data from an online survey from a German sample which found that most respondents gambled less or stopped gambling during lockdown, while only a minority reported increased gambling behaviour (Georgiadou et al., 2022). These appear to be particularly vulnerable individuals (Håkansson, 2020; Hodgins & Stevens, 2021; Price, 2020). However, while the number of posts in the problem gambling subforum decreased during the first lockdown, the number of posts in the online gambling subforum increased. It is not clear whether the shift from terrestrial to online gambling reflects a reduction in problem gambling, or whether people who gamble predominantly online seek less help. Online gambling was found to be more likely among persons who gamble with pre-existing problems (Gainsbury et al., 2015) and among problem gamblers, help-seeking in online support groups or discussion boards was lower for online compared to terrestrial gamblers (Hing et al., 2015).

Addiction and COVID-19 have been described as colliding pandemics (Dubey et al., 2020; Volkow, 2020). The social distancing and lockdown measures create special challenges to individuals at risk. In the case of gambling, the closure of arcades and casinos may have led to a shift from terrestrial towards online gambling. In contrast to the terrestrial arcade, where an arcade supervisor carries out age checks and is required to identify problem gambling behaviour, online gambling is not subject to such checks. This may pose a special threat to individuals at risk for gambling addiction. The current analyses help to identify effects of the lockdown periods on gambling behaviour. These potentially detrimental effects on mental health should be monitored and may require special public health measures.

### Limitations, Strengths and Ethical Considerations

A major limitation of our approach is that the degree to which changes in posting behaviour reflect changes in actual gambling behaviour is unclear. A higher posting frequency, a higher mean post length and shorter reply latencies in the online gambling subforum indicate that users write more and longer posts about online gambling and respond faster to these topics. While this may reflect increased online gambling behaviour, it may only indicate changes in communication about the subject matter. Similarly, changes in posting frequency, post length and the reply latencies in the problem gambling subforum indicate changes in the communication on problem gambling, but do not necessarily represent changes in actual problem gambling behaviour or changes in the prevalence of disordered gambling among forum users. However, since gambling venues were closed during the lockdown, changes in actual gambling behaviour could not be systematically investigated on site. Systematically measuring online gambling behaviour was also hardly possible, since users typically visit many different online gambling platforms. Measurements would then have to be based on self-reports, which have limitations such as social-desirability bias (Krumpal, 2013) and recall bias (Bradburn et al., 1987). Specifically for gambling, it further appears that self-reported gambling behaviour is not reliable due to misestimation of losses (Heirene et al., 2021). However, the participation in online gambling communities is linked to problem gambling (Sirola et al., 2018, 2019) and recently, there have been first successful attempts to predict actual mental health from social media posts on Facebook (Merchant et al., 2019). Still, linking actual gambling behaviour to behaviour in online gambling communities and to self-reports needs to be elucidated by future research. Finally, the users of the forum are likely not representative for the population of all gamblers. In a Finnish sample, more than half of the people who visit online gambling communities at least once were individuals at-risk or likely pathological gamblers (Sirola et al., 2018), while the proportion is markedly lower in the general population of gamblers (Floros, 2018).

A strength of our approach lies in its high ecological validity. We examined actual posting behaviour in a real-life setting, circumventing shortcomings of self-reports such as the social-desirability and recall bias(Bradburn et al., 1987; Krumpal, 2013). Posts are not self-reports in a strict sense, however, how people communicate in such forums depends on the degree of perceived anonymity and identifiability of users, resulting in different norms with regard to content and conversational style. This is formulated in the Social Identity Model Of Deindividuation (SIDE) model (Reicher et al., 1995). Further, there appears to be a general tendency to present oneself idealised online, which may also be reflected in the style of communication in the forum (Zimmermann et al., 2022). These issues may be an outlook for more specific analyses.

An advantage of using data scraped from public web forums is that large datasets may be collected without researchers’ interference, that is, there are no effects of observation. However, this also raises important ethical issues. Eysenbach and Till (2001) emphasise the importance of “perceived privacy” in an online community. If, for instance, posts can only be viewed following registration, this suggests that users might perceive their contributions as occurring in a private space (Eysenbach & Till, 2001; Landers et al., 2016). Likewise, if providers put measures in place to restrict data access, researchers should not circumvent these measures (Landers et al., 2016). We have followed these guidelines and collected data that were publicly available and did not bypass any technical barriers in doing so.

### Perspectives

In addition to a quantitative analysis of posting behaviour, a qualitative analysis of post content may yield further insight into gambling behaviour and gambling-related issues among gamblers in follow-up studies. For instance, many of the initial posts from the problem gambling subforum contain detailed descriptions of gambling-related problems and mental health issues, while reply posts included tips on coping with gambling addiction and discussions about coping strategies and treatments. Automated text analysis and computational methods may be useful tools for identifying user topics and signs of problematic gambling behaviour (Haefeli et al., 2015; Hwang et al., 2020; Maupomé et al., 2021; McGarry & McDonald, 2017). This may facilitate player protection through early detection of gambling addiction risk, complementing human assessment, and on the part of gambling operators, prioritising customer contacts based on risk assessment (Haefeli et al., 2015).

## Data Availability

The data underlying the study were scraped from a large German online gambling forum and were openly available before the initiation of the study. All data with the exception of data related to individual user profiles and user names are openly available at https://osf.io/rwym3/.

## Appendix A

See Table 3.

**Table 3:** Casino closure periods in the federated states of Germany

	1st lockdown period		2nd lockdown period	
State	Start	End	Start	End
Baden-Württemberg	17/03/2020	10/05/2020	02/11/2020	07/06/2021
Bavaria	17/03/2020	10/05/2020	02/11/2020	07/06/2021
Berlin	14/03/2020	01/06/2020	02/11/2020	18/06/2021
Brandenburg	18/03/2020	27/05/2020	02/11/2020	11/06/2021
Bremen	18/03/2020	26/05/2020	02/11/2020	31/05/2021
Hamburg	15/03/2020	26/05/2020	02/11/2020	04/06/2021
Hessen	18/03/2020	14/05/2020	02/11/2020	10/06/2021
Mecklenburg-Western	18/03/2020	14/06/2020	02/11/2020	07/06/2021
Pomerania Lower Saxony	17/03/2020	24/05/2020	02/11/2020	31/05/2021
North Rhine-Westphalia	16/03/2020	10/05/2020	02/11/2020	28/05/2021
Rhineland-Palatinate	18/03/2020	26/05/2020	02/11/2020	02/06/2021
Saarland	18/03/2020	03/05/2020	02/11/2020	31/05/2021
Saxony	19/03/2020	14/05/2020	02/11/2020	14/06/2021
Saxony-Anhalt	18/03/2020	27/05/2020	02/11/2020	25/05/2021
Schleswig-Holstein	14/03/2020	17/05/2020	02/11/2020	31/05/2021
Thuringia	18/03/2020	12/05/2020	02/11/2020	02/06/2021
Overlap	19/03/2020	03/05/2020	02/11/2020	25/05/2021
